# A Formative Assessment of Social Determinants of Health Related to Early Childhood Caries in Two American Indian Communities

**DOI:** 10.3390/ijerph18189838

**Published:** 2021-09-18

**Authors:** Kristan Elwell, Carolyn Camplain, Christine Kirby, Katharine Sanderson, Gloria Grover, Gerlinda Morrison, Amy Gelatt, Julie A. Baldwin

**Affiliations:** 1 Department of Health Sciences, Northern Arizona University, Flagstaff, AZ 86011, USA; kristan.elwell@nau.edu (K.E.); julie.baldwin@nau.edu (J.A.B.); 2 Center for Health Equity Research, Northern Arizona University, Flagstaff, AZ 86011, USA; Katharine.Sanderson@nau.edu (K.S.); amy.gelatt@nau.edu (A.G.); 3 Hopi Department of Health and Human Services, Kykotsmovi, AZ 86039, USA; glogrover11@gmail.com; 4 Little Big Horn College, Crow Agency, MT 59022, USA; morrisong@lbhc.edu

**Keywords:** early childhood caries (ECC), social determinants of health (SDH), American Indian communities

## Abstract

In the United States, children from diverse ethnic groups and those with low socioeconomic status are at a significantly increased risk for early childhood caries. Despite the efforts focused on decreasing early childhood caries in American Indian (AI) populations, these children have the highest incidence of dental caries of any ethnic group, with four times the cases of untreated dental caries compared to white children. This qualitative formative assessment was conducted in two AI communities. Semi-structured interviews (*n* = 57) were conducted with caregivers and providers to understand the social and community contexts in which oral health behaviors and practices occur from the perspective of the caregivers, oral health care providers, and social service providers in the communities. The analysis was informed by the social determinants of health framework. The key social determinants of pediatric oral health relevant to our study communities included limited access to: oral health promoting nutritious foods, transportation for oral health appointments, and pediatric specialty care. This formative assessment provided locally and contextually relevant information to shape the development of an oral health clinical trial intervention to address early childhood caries in these two communities.

## 1. Introduction

Early childhood caries (ECC) is the presence of one or more decayed, missing, or filled teeth (dmft) in any primary tooth in a child of less than 6 years of age [1]. Even though oral health guidelines suggest that oral health care should begin when the first tooth erupts or by age one, few children receive oral health care at this age [2]. In addition, due to the chronic nature and delayed onset of pain with untreated dental caries, children are often not seen for dental treatment until it is too late to avoid severe symptoms and intensive treatment [3].

ECC prevalence is high in the US, where approximately 23% of children aged 2–5 years have dental caries [4]. In the US, children from diverse ethnic groups are at significantly increased risk for caries as compared to white children [5]. American Indian (AI) children have the highest incidence with 52.0% of children under the age of five experiencing ECC nationally [6].

A child’s chance of developing ECC is influenced by biological, behavioral, and social and structural factors related to the social determinants of health (SDH) [7]. The social determinants of health are defined as “the conditions in which people are born, grow, work, live, and age, and the wider set of forces and systems shaping the conditions of daily life … include[ing] economic policies and systems, development agendas, social norms, social policies and political systems”. The SDH relevant to children’s oral health [7] include low oral health literacy, limited access to employment opportunities, oral health care, oral health resources (dental products, clean water), healthy foods, and social support [7]. Interventions targeting behavioral, social, and structural risk factors impacted by the SDH should provide a comprehensive and holistic approach for the reduction of ECC. This formative assessment aimed to identify the health and social factors relevant to the design and implementation of an intervention to reduce ECC in two AI communities. 

The objectives of this community-driven formative assessment were to partner with one Southwestern tribe and one Plains tribe to conduct an assessment of oral health beliefs and practices in the communities. This assessment aimed to identify the priorities, barriers, and resources of each community to address the oral health needs of pregnant women and young children. The study objectives were to: assess providers’ and caregivers’ oral health knowledge and practices; identify potential barriers and facilitators to successful implementation of the intervention; determine how to adapt the intervention to ensure its sustainability; understand community values and practices, psychosocial, social and structural resources and needs; and examine mothers’ understandings of promoting oral health in young children.

The research team had long-standing, working relationships with the two tribal communities and was invited to assist in developing this study and the subsequent intervention. Both of the tribal communities have similar contextual characteristics, including being located in rural and geographically remote regions, having major concerns regarding oral health and ECC prevalence, being relatively small tribal communities located in economically depressed areas, and having limited resources available to address the oral health needs. Even with these similarities, these communities are both socially and culturally unique and comparisons between the tribal communities cannot be drawn. 

## 2. Materials and Methods

**Figure 1:**
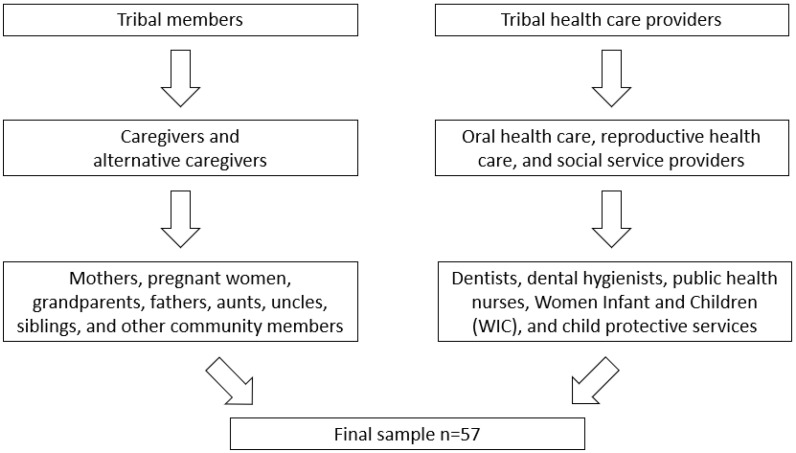
Flow chart of study participants.

This qualitative study was developed using a social determinants of health framework [8]. This dynamic framework recognizes the interaction between structural and intermediary determinants in shaping health outcomes whereby structural factors lead to determinants such as access to oral health services, transportation, and social support. The framework allows for the examination of the intersection of the behavioral, social, and environmental factors that contribute to ECC by contextualizing oral health practices in the daily lives of AI women during pregnancy. Semi-structured interviews (*n* = 57) were conducted with caregivers and providers in two AI communities. Please see Figure 1 for the flow chart of study participants. Table 1 shows the breakdown of provider and caregiver interviews. The social determinants of health framework informed our approach to understanding the social context of oral health for young children in the study communities.

Between February and May 2019, the research team conducted in-depth, semi-structured interviews with health care providers and caregivers at each study site. The health care providers included oral health care, reproductive health, and social service providers as identified by the tribal communities involved. The study sample included dentists, dental hygienists, public health nurses, Women Infant and Children (WIC) employees, and child protective services employees. The health care and social service providers were recruited through professional affiliations using purposive and snowball sampling techniques to ensure that participants with insight about oral health and women’s prenatal and postnatal needs were included in the study sample. The inclusion criteria for caregivers were mothers, pregnant women, and alternative caregivers (including grandparents, fathers, aunts, uncles, siblings, and other community members) who were members of the tribal communities included in the study. The study included 20 caregivers (mothers or pregnant women) and 7 alternative caregivers. The caregivers were recruited through local social service organizations and professional affiliations utilizing purposive and snowball sampling techniques.

The study was conducted according to the guidelines of the Declaration of Helsinki, and approved by the Institutional Review Board (IRB) of Northern Arizona University (protocols 1309361 and 1396150; 09-26-18 and 04-26-19). Informed consent was obtained from all subjects involved in the study. 

The interview questions for providers assessed their perceptions of the caregivers’ oral health knowledge and practices, identified potential barriers and facilitators to successful implementation of the intervention, and examined how to adapt the intervention and ensure its sustainability. The interviews with pregnant women, new mothers, and alternative caregivers examined these same themes as well as values and practices; psychosocial, social, and structural resources and needs; and the mothers’ understandings of oral health. The interviews were conducted until a thematic saturation was reached and informants no longer reported new information. The interview transcripts were analyzed using an inductive approach to capture important themes or recurring issues. 

### Analysis

The interviews were audio recorded and transcribed verbatim by a transcription company (Rev.com accessed on 17 September 2021). The data were analyzed using applied thematic analysis techniques [9] and a constant comparative analysis [10]. The constant comparative method is a method in which responses to questions are compared among the key informants to refine key concepts. The analysis was designed to explore variations in how key concepts are discussed, examine interrelationships, and integrate them into a model of provider and caregiver perspectives on the factors that influence mothers’/caregivers’ knowledge, beliefs, and practices related to oral health in young children.

We employed an iterative process of interviewing and thematic coding to guide our analysis. First, the research team developed an initial codebook. Three team members (KE, KS, CK) met several times to compare codes until an agreement was reached about the meaning and application of each code prior to coding the remaining dataset. This method is intended to improve interrater reliability while still allowing emergent themes to be recognized and incorporated into the analysis. The transcripts were then coded with the NVivo qualitative data analysis software 11 on a secured server using an applied thematic analysis technique.

After reaching saturation and having coded the entire dataset, we conducted a content analysis of selected research questions to determine which barriers and supports were most common within each community. We asked the following questions of the data:

(Oral health knowledge) What do caregivers know about the relationship between oral health and dietary practices?

(Oral health attitudes) What do caregivers think about oral health in young children? Is oral health a priority for new mothers in these communities? 

(Barriers) What are the major barriers to accessing oral health care for children at the individual, provider, community, and clinic levels?

(Issues) What are the major social and structural issues caregivers face which can prevent positive oral health in children?

(Supports) What are the major supports for promoting oral health care for children at the individual, community, and clinic levels?

The major themes from the interviews informed the adaptation of the health education materials for local and contextual relevance, and cultural appropriateness (we define cultural appropriateness as the saliency and relevance of oral health and prenatal/postnatal guidelines to the day-to-day lives of AI pregnant and postpartum women living in the participating communities, recognizing that members of AI communities do not subscribe to attitudes and beliefs with uniformity).

## 3. Results

The analysis revealed multiple factors influencing young children’s oral health in the study communities. This paper will focus on three major themes from the analysis: oral health knowledge and values, barriers to children’s oral health, and supports for children’s oral health. The perspective of oral health care providers, community members, and caregivers are presented below. In addition, Table 2 and Table 3 provide an overview of caregiver priorities and provider perspectives on common barriers to caring for children’s teeth at home, respectively. 

### 3.1. Oral Health Knowledge and Attitudes

#### Provider and Community Perceptions of Caregivers’ Oral Health Knowledge

When asked to describe the typical oral health of young children in their community, oral health providers described the state of the children’s oral health as “very poor”. Several providers described the children’s oral health as an “emergency”, with young children often seeking care only when they are in pain. In many cases, children needed treatment for more acute oral health conditions which require extractions. Many described these dental emergencies as occurring before a child reached the age of 2. Several providers expressed a strong commitment to addressing ECC in their community and were dismayed by the “lack of progress” being made to minimize ECC despite a significant clinical effort.

*“We do see quite a few kids aged one to three. I would say it’s poor, you know, their oral hygiene habits, care. Yeah, I, I would say we probably ended up sedating over half of them to do treatment on them.”*

(Dental provider)

When asked to comment on the key challenges to ECC in their communities, many of the providers cited a lack of parental knowledge and understanding regarding why early childhood oral health is important (33%); how to care for a child’s teeth (30%); or the impact of sugar consumption on children’s oral health (23%).

Similarly, many of the caregiver participants (44%) perceived that a major barrier to caregivers’ ability to care for their children’s teeth stemmed from not knowing (1) how to care for children’s teeth, (2) how often they should brush their children’s teeth, or (3) why it is important. Some of the participants felt that parents do not think baby teeth are important because “they don’t know how the baby teeth affect the permanent teeth” or think that baby teeth do not matter because “they’re just going to fall out anyway”. 

### 3.2. Caregiver Oral Health Knowledge

Although oral health knowledge was cited as a major barrier by the oral health providers, the caregivers were knowledgeable about several aspects of children’s oral health, including when to start caring for a child’s teeth and when to start taking a child to the dentist. Fifty-five percent of the caregivers interviewed believed that caregivers should start taking children to the dentist before they get teeth, when their teeth start to erupt (22%), or around 1 year of age (22%). Forty-eight percent of the caregiver participants said that caregivers should start caring for a child’s teeth immediately after birth or before they start getting teeth.

As one participant stated,

“*I remember reading a pamphlet somewhere or something that you have to have a check up on your kids at maybe 3 or 6 months even though they don’t have teeth, just to make sure their gums are healthy*.”

(Caregiver)

### 3.3. Diet and Oral Health

While almost a quarter of the providers (23%) reported that parents do not understand the relationship between diet and oral health, most of the caregivers (85%) interviewed clearly understood the oral health impact of sweet foods and beverages on the risk of tooth decay. About 33% of parents discussed bottle rot and the problems that frequent bottle feeding and putting sugary drinks in bottles can cause.

“*Well, I made her quit bottle, when she turned two. She quit bottle, ‘cause I thought that was where the cavities came in, ‘cause they want milk, or they want the juice or pop in a bottle…. We didn’t have a problem with her cavities so much after I had her quit the bottle*.”

(Caregiver)

While the importance of restricting sweet drinks and foods was commonly known among the caregivers in the study, many participants suggested this can be a challenge. The children’s consumption of sugary foods and drinks was influenced by factors at the family and community level. At the family level, the participants suggested that providing sweet drinks is sometimes utilized by parents to assuage children when they are “fussy” and by the grandparents and other members of the extended family as a means to bond with young children. For example, some of the caregivers (19%) reported that they or parents they know often “give in” to a child “nagging” or give in to sugary treats to appease their children. As one participant describes parents giving in to children’s “nagging”,

“*I see a lot of mothers ‘here, drink this’ and it’s a bottle full of Gatorade, or a bottle full of pop. Anything, sometimes just to keep your kids quiet*.”

(Caregiver)

### 3.4. Sources of Caregiver Oral Health Knowledge 

The caregivers described strong family influences when learning about how to care for children’s oral health. In both of the study communities, the extended family is often directly involved in the children’s caregiving, including when parents are learning how to care for children’s teeth. When asked where most of the parents learn how to provide oral health care in young children, 63% of the caregivers reported learning about how to care for their children’s teeth through family members, such as parents, aunts, grandmothers, or siblings with children. While most of the caregivers learned about oral health through family, 56% of the caregivers noted that they often received oral health education through local community maternal and child health programs, such as WIC and the tribal health department’s Community Health Representative (CHR) programs. Two of the caregivers stated they received their oral health information from the dentist. 

The critical support provided by the elders was moderated by the fact that some of the caregivers (33%) found it challenging to control what other caregivers or family members fed their child and struggled to get other family members and caregivers to support the parents’ wishes regarding the children’s diet and toothbrushing. The community members described the importance of demonstrating respect for the elders and other family members when managing children’s consumption of sweets by not turning down food when offered. 

Recognizing the need to limit sweets for her child, one caregiver discussed the challenges of reinforcing this health behavior when family members are present.

“*And then my parents give in too, so they get more intake of sugar than they should be. It actually affects it a lot. I know that what they eat and drink has a lot to do with their oral health. Even with my boyfriend’s kids I always talk to them about not giving them candy cause that something their aunties and grandmas do because you know they’re going to do it anyway. There’s no way to stop them, they’re going to find a way to give them pop and candy but as a parent you see them the majority of the time so if you don’t give it to them they’re only going to have it from like them*.”

(Caregiver)

### 3.5. Barriers to Children’s Oral Health Promotion

#### Household Level Barriers: Prioritizing Oral Health

Fifty three percent of the providers interviewed perceived that parents in the community do not prioritize their children’s oral health. The providers perceived that parents do not think oral health is important, are not interested in oral health, or simply “don’t care”. Some of these discussions were tied to larger issues of child neglect or poor parenting practices. The same percentage of providers (53%) also recognized that parents do not have time to care for their children’s oral health because other concerns take priority.

While 44% of the caregivers cited apathy as being a barrier to parental care of children’s oral health, 56% of the caregivers discussed the frequency with which parents struggle with multiple competing priorities that interfere with the parents’ ability to prioritize oral health for their young children. Many of the caregiver participants discussed being busy with “other things” or not having time to take care of their children’s teeth at home (56%) or take them to the dentist (30%). Because of other responsibilities, the caregivers explained that sometimes they are tired and do not have the energy to take care of their child’s teeth after a long day at work.

“*I kind of feel guilty about like, ‘Well, I brush my teeth every day, why can’t I brush my son’s every day?’ But it’s just, it’s hard, especially when you’re tired and…you’ve been up since 5:30*.”

(Caregiver)

Even with many competing priorities, the caregivers discussed several reasons why they value the oral health of their child. When asked why their child’s oral health is important to them, the caregivers commented that preventing more serious oral health problems, preventing problems with the child’s appearance, and preventing pain were reasons they valued maintaining their child’s oral health. The most commonly discussed reason for taking care of children’s teeth was wanting to prevent the child from developing more serious problems in the future (52%). This included preventing the child from losing teeth (48%), getting cavities (22%), or developing other health conditions (11%). 

About 33% of the caregiver participants discussed appearance as being a source of motivation to take care of children’s teeth. The participants reported wanting their children to have pretty teeth (15%) and confidence in their appearance (41%). Some also mentioned not wanting their children to have silver caps on their teeth (15%). Two participants (7%) discussed not wanting their children to experience the stigma associated with poor dental hygiene. Similarly, about 33% of the caregiver participants discussed wanting to prevent their child from experiencing pain associated with poor oral health. This was often in relation to painful procedures that may be necessary if oral health problems require clinical treatment, such as shots of Novocain or painful surgeries.

“*Here on the reservation I see a lot of the tooth decay, and I think the reason is why I switched my son to a cup right away is because I was scared of the bottle rot, they told me that it would start, the two front teeth would start getting a dent in them, so I really don’t want my son to have any cavities. But I know it’ll happen*.”

(Caregiver)

### 3.6. Community- and Structural-Level Barriers: Reliable Transportation, Access to Health Promoting Foods, and Access to Specialty Oral Health Care

The SDH, such as the policies, practices, and social conditions that indirectly shape caregiver’s oral health practices, emerged as barriers to children’s oral health. The most common barriers included finding consistent transportation and affordable, health-promoting food. Both of the communities mentioned the lack of fruits and vegetables in local stores and the greater expense of these foods in comparison to sugary foods and drinks. 

Forty-five percent of the caregivers and most of the providers (67%) cited transportation as a primary barrier to accessing oral health services, either for themselves or other community members. Both of the study communities are situated within rural regions where reliable transportation is limited. To address this gap, one study community provides transportation assistance programs. At both of the study sites, a lack of gas money to get to appointments was cited by 19% of caregiver participants as a reason that patients miss appointments.

“*Sometimes when [my dad] is busy at work, he can’t usually let me use a ride so then I have to reschedule*.”

(Caregiver)

“*I think a lot of my peers struggled with not having a reliable transportation and they either can’t get a ride or they depend on the local transit that we have here*.”

(Caregiver)

The caregivers and providers discussed several barriers to improving their child’s dietary practices to promote positive oral health. Seven percent of the caregivers shared that they often buy convenience foods rather than healthy foods due to a lack of time or energy to prepare healthier foods. Eleven percent of the caregivers discussed the fact that food stores often charge higher prices for food such as fresh fruit, vegetables, and proteins such as meat and cheese. Thirteen percent of the providers interviewed noted that reservation communities are often “food deserts”, which contribute to poor oral health.

“*It’s all the sugary stuff. I mean, I’m not saying don’t cut out sugar from your, but moderate it, and make sure that you brush. Because my kids, especially now that I have this hectic schedule, I can’t feed them healthy home-cooked meals as much as I want to. And so, they have no choice but to pop something in the microwave, or heat something up, like pizza, or snack on chips and stuff, just because, easier access. We’re on the go*.”

(Caregiver)

“*I think apple juice. He drinks a lot of apple juice and Gatorade. He eats Cheetos. So he kind of likes, I try to keep him away from junk food, like chips and just whatever he wants, I am trying to control it… But even, at the fast food places, seems like the healthier drinks cost more*.”

(Caregiver)

Given the high rates of ECC among AI children, access to specialty care is critical for children who need immediate extractions due to caries severity. Severe decay that is left untreated can quickly progress to an infection of the inner root of the tooth and spread to the sinuses or other areas of the head and neck, causing damage to other teeth, tissues, or in rare cases, lead to death [11]. These cases must be treated immediately and often require the use of general anesthesia for young children, which is costly and poses a higher risk to the child, demanding the skill of a trained pedodontist and anesthesiologist. In this study, we found that a lack of pediatric dentistry was a common concern for families in both of the study communities. In one community, accessing a pediatric dentist involves a 2–3-h drive. This added burden when time and money are limited creates additional financial challenges for families who have children in need of oral surgery. Most of the providers noted the limited local oral health services available to young children in need of pediatric oral health care (*n* = 18)**.** Commenting that they frequently need to refer patients to distant facilities to obtain oral health services not available at their facility, the providers suggested that following up with patients that have received care elsewhere contributes to a lack of continuity in care.

### 3.7. Supports for Children’s Oral Health Promotion

#### Household Level Supports: Family Support

Managing the influence of family members posed a challenge when trying to minimize children’s consumption of sweets. However, when asked what factors promote positive oral health in young children, 22% of the caregiver participants stressed the pivotal role of family relationships and close-knit communities as integral to the well-being of young children. Strong family and community relations are a key source of support and resilience within both of the study communities, providing a unique resource to new parents. Given that many family and community members often guide new parents in children’s caregiving practices, including children’s oral health, these close relationships can help new parents reinforce oral health promoting practices. Eighty-one percent of the caregivers interviewed live in multigenerational households. This includes participants who reported living with people other than their partner and children, and commonly included siblings, parents, grandparents, and in-laws. These participants placed significant value on the support provided by members of the extended family who live in the same household. Such support included helping with caregiving, transportation to appointments, and meal preparation. Furthermore, 33% of the caregiver participants specifically discussed the importance of having the whole family help support and reinforce positive oral health habits, including setting a good example or directly assisting parents when caring for children’s teeth. 

The caregiver below discusses the caregiving support she receives within her multigenerational home:

“*Well, my whole family. My mom helps me in the morning and then my cousin… Well, there’s my cousin, my grandma and aunt, they all pitch in and help watch him, of course*.”

(Caregiver)

One caregiver describes how her sister, who lives in the surrounding area, is engaged in her children’s oral health:

“*She’ll tell me too, are you checking his teeth? All the kids when they go see her, “Are you taking care of their teeth?” Yes, I am. She encourages me a lot*.”

(Alternative caregiver)

### 3.8. Community-Level Supports: Local Resources 

As discussed earlier, there are many community programs and organizations that provide information and support for parents and caregivers regarding children’s oral health. WIC was the most commonly mentioned program for support with oral health among the caregivers (37%). 

Forty percent of the providers also discussed community initiatives that support oral health. These included Head Start, local wellness clinics, CHR programs, and WIC. The providers commented that raising awareness of the importance of oral health through existing community-based programs, such as school outreach programs and health fairs, can be an effective strategy to promote children’s oral health.

“*With my daughter, for example, when she would take her to her Well Child checks the doctor would kind of tell her that she needs to clean inside the mouth and give her that finger swab thingy… It’s just education from somebody, a professional, and just drilling it into their head to make sure that it has to be done*.”

(Caregiver)

Many of the providers interviewed also recognized the strength and resilience that comes from being a close-knit community (*n* = 6). Close social bonds create a strong sense of community pride and hope for the future. When asked, “what factors within the community facilitate positive general health or resilience”, one provider said, “Self-esteem. A sense of community. A sense of family…I think having a sense of hope and a sense of some self-esteem and that there’s a future out there, that makes it possible for people to take measures to increase health and to do that prevention kind of stuff.” (Social service provider). 

Some of the providers also mentioned passionate people working and living in the community as a source of strength and resilience. Many people also have a personal connection with their health care or dental care provider, which makes people more willing to ask for help and or take the initiative to take care of their health (*n* = 3).

“*I think it would be the strong community relationships just because we do have people that know each other on a first name with their providers, I love it… I think everyone just appreciates that and they’re more willing, if they feel comfortable then they’re more willing to ask for the help or go out and get things done*.”

(SSPROV02H)

“*Actually, cohesive family arrangements. Mainly the grandparents and great grandparents getting involved in the community. That is a very strong point for them…we are very thankful for the whole community structure*.”

(Dental provider)

### 3.9. Linkages between the Formative Assessment and Subsequent Oral Health Intervention

The results from this formative assessment were applied to a larger oral health clinical trial designed to be locally and contextually relevant and attentive to the social determinants of health applicable to the lives of AI pregnant women and new mothers. This bundled, best practices intervention includes delivery by the Community Health Representatives (CHRs), the use of motivational interviewing (MI) techniques, the provision of fluoride varnish, and an emphasis on providing oral health education to pregnant women and new mothers well before their child reaches the age of three years old. The oral health intervention arm will be compared to a healthy lifestyle intervention arm that will provide participants with general maternal and child health education.

The research team utilized the findings above to begin revising and culturally tailoring both the oral health and healthy lifestyle comparison intervention materials and processes. The formative assessment results allowed the research team to determine the content of the health education material and the best way to present the material to the potential participants. The specific topics added to health education materials included: recognizing support from the extended family in children’s caregiving; strengthening caregiver strategies for promoting children’s oral health (modeling, making brushing fun); honoring local traditions; reinforcing existing oral health and MCH resources in the study communities; and incorporating locally relevant foods. 

## 4. Discussion

ECC remains a significant public health problem in many AI communities across the United States. To better understand the impact of ECC in two AI communities, this study examined the knowledge, attitudes, and beliefs related to ECC, and the SDH that intersect with the main barriers and supports impacting ECC in these communities. The results were used to inform the development of a locally and contextually relevant oral health intervention that targeted women during pregnancy and in the postnatal period.

When examining parents’ knowledge about young children’s oral health, we found a gap between the provider perceptions of caregiver knowledge of the oral health needs of young children and the caregiver’s knowledge of several aspects of children’s oral health. This is the first study, to our knowledge, that has found a gap in patient–provider perspectives related to AI children’s oral health, but it is not likely to be unique to the AI population based upon the experience of the dental research team members who see this in their practice for the caregivers of children from all backgrounds. The literature suggests [12,13] that differences between patient and provider perspectives regarding disease management are not uncommon and are rooted in educational and socioeconomic differences, and therefore are not necessarily unique to AI children. Due to the high rates of ECC/disease in the AI population, however, it is possible that misconceptions are more widespread among AI populations. However, to the best of the authors’ knowledge, there are no data or publications to support this. It is possible that differences between the patient and provider perspectives are more likely to correspond with a lack of resources, access to care, and the social and structural challenges related to low socioeconomic status that is common among high-risk groups. It is also possible that the geographic isolation of many AI populations plays a role.

The caregivers had a good understanding of children’s oral health. Generally, the caregivers were aware of the need to seek oral health care before children have their first tooth. They were also aware of the need to limit sweets from children’s diets and to limit sweet drinks in a baby’s bottles to prevent bottle rot. We found that the caregiver’s knowledge and attitudes toward children’s oral health depended on what they learned from family members, local social service programs, and prior experiences with oral health when the caregivers were children themselves. Only two of the caregivers stated they received their oral health and dental information from local dentists or oral health providers. This is not uncommon among new parents. The literature on caregiver knowledge of young children’s oral health care needs suggests that although they are aware of many aspects of oral health, many parents are not aware of the importance of baby teeth [14,15]. 

Nearly half of the providers and community members interviewed cited parental apathy in caring for their child’s teeth. However, the caregivers noted that competing priorities often interfere with a parent’s ability to care for their child’s teeth. Household management, work, family, and sociocultural responsibilities, and in some instances, family and relationship challenges, impacted the daily lives of caregivers in this study. 

The caregivers were clear, however, on why they place value on their child’s oral health. A key motivation for promoting children’s oral health was to prevent pain and promote a positive appearance. The caregiver discussion of the importance of having a positive appearance revolved around wanting their child to have the confidence to show their smile, not being embarrassed by poor oral health, and wanting to protect their child from the stigma associated with poor oral health. This may be one reason why a child’s appearance was important to those community members who participated in this study.

Beyond the household level factors affecting children’s oral health, community and structural factors are at play in these communities. Limited transportation prevents many of the caregivers from taking their child to dental appointments. Further, access to healthy foods as an alternative to sweets and other foods that contribute to ECC is challenging when communities are characterized as “food deserts”. In a nationally representative study, researchers found that children from households with low or very low food security had significantly higher caries prevalence [16]. Promoting children’s oral health when access to nutritious foods is both limited and expensive can be challenging in any community. Yet, affordable healthy food is critical to children’s oral health promotion and must be addressed at the national policy level and at the highest levels of tribal government.

While many barriers to children’s oral health promotion were cited by the study participants, there are also many opportunities to improve children’s oral health. First, educating family members from each generation may be one way to maximize the opportunity and reinforce positive oral health in AI communities. As a key source of oral health knowledge, family members can play a major role in supporting new parents to provide oral health care for their young children. However, parents need access to culturally tailored information on how to negotiate with family members who share the caregiving responsibilities. For example, sharing new knowledge of the oral health practices in young children with family members is critical, especially when family members provide sweet foods and sweet drinks when bonding with children. 

This study uncovered unique challenges within the two tribal communities in this study; however, it was not without its limitations. One limitation is that this study only involves two tribal communities and may not be generalizable to other tribal communities in the US. Each of the 574 federally recognized and 63 of the state recognized tribal communities in the United States may face similar but also unique challenges. This study does not represent the AI community as a whole. The goal of this particular study was to culturally tailor an intervention for two tribal communities; therefore, it was not designed to be generalizable. The study sample was small; however, it was on par for a qualitative study of this nature and we did reach theoretical and topical saturation with this sample size. This study utilized purposive and snowball sampling. Qualitative studies with sample sizes such as ours are not intended to demonstrate causality, nor be generalizable to other communities [17]. Finally, the study team recognizes that while the literature demonstrates the causal role of SDH in contributing to health status, there is little research documenting the effectiveness of reducing health inequalities by socioeconomic status through public health interventions [18].

## 5. Conclusions

To build upon the existing health promotion resources within the community, partnering with local maternal and child health programs that offer critical services to pregnant women and new mothers is an additional strategy to reinforce the significance of young children’s oral health at a time when pregnant women might be more focused on monitoring their own health and preparing to take care of their new baby.

While there are many strengths within both of the study communities, there are also opportunities to promote oral health in these tribal communities. Recognizing the role of the SDH in children’s oral health care can support AI caregivers in making health-promoting decisions about their child’s oral health and ensure oral health equity among AI children. Future research and oral health education for parents should consider utilizing a family-centered health education model where caregivers from multiple generations participate in oral health education for the entire family well before a child is born.

## Figures and Tables

**Table 1:** Provider and caregiver interviews.

	Study Site 1	Study Site 2	Total
Social service providers	7	2	9
Reproductive health providers	5	1	6
Oral health providers	5	10	15
Caregivers	10	10	20
Alternative caregivers	5	2	7
Total	32	25	57

**Table 2:** Caregiver priorities.

Caregiver Priorities	Total *n*
General health (family, self, child)	*n* = 16
Financial concerns	*n* = 6
Physical safety (this included staying safe in one’s community)	*n* = 6
Mental health (this included being happy, being mentally healthy and abstaining from drugs and alcohol)	*n* = 6
Basic needs (staying warm, having good food, a place to sleep, clean)	*n* = 5
Prioritizing children	*n* = 4
Child’s education	*n* = 2
Oral health	*n* = 2
Family dynamics	*n* = 2

**Table 3:** Provider perspectives on common barriers to caring for children’s teeth at home.

Provider Perspectives on Common Barriers to Caring for Children’s Teeth at Home	Total *n*
Lack of knowledge	*n* = 19
Knowledge on why child oral health is important:	*n* = 10
Knowledge about how to care for a child’s teeth:	*n* = 9
Knowledge on between diet or feeding practices and oral health:	*n* = 7
Parents do not prioritize oral health	*n* = 16
Have bigger concerns than oral health	*n* = 16
Parents missing child’s oral health appointments	*n* = 13
Diet	*n* = 9
General parenting problems	*n* = 5
Poor oral health self-care practices among caregivers	*n* = 5
Fear	*n* = 3

## Data Availability

The data presented in this study may be available on request from the corresponding author. The data are not publicly available due to IRB guidelines and ownership of data.

## References

[1] Vastine A., Gittelsohn J., Ethelbah B., Anliker J., Caballero B. (2005). Formative research and stakeholder participation in intervention development. Am. J. Health Behav..

[2] WHO Social Determinants of Health. https://www.who.int/health-topics/social-determinants-of-health#tab=tab_1.

[3] Urahn S., Snyder A.G.S., Hoppock J. (2011). The State of Children’s Dental Health: Making Coverage Matter.

[4] Moyer V.A. (2014). Prevention of dental caries in children from birth through age 5 years: US Preventive Services Task Force recommendation statement. Pediatrics.

[5] Anil S., Anand P.S. (2017). Early childhood caries: Prevalence, risk factors, and prevention. Front. Pediatrics.

[6] Phipps K.R.R.T., Mork N.P., Lozon T.L. (2019). The Oral Health of American Indian and Alaska Native Children Aged 1–5 Years: Results of the 2018-19 IHS Oral Health Survey.

[7] da Fonseca M.A., Avenetti D. (2017). Social determinants of pediatric oral health. Dent. Clin..

[8] Solar O., Irwin A. (2010). A Conceptual Framework for Action on the Social Determinants of Health.

[9] Trotter Ii R.T. (2012). Qualitative research sample design and sample size: Resolving and unresolved issues and inferential imperatives. Prev. Med..

[10] Bernard H.R., Ryan G.W. (2009). Analyzing Qualitative Data: Systematic Approaches.

[11] Dentistry A.A.o.P. (2019–2020). Policy on Early Childhood Caries (ECC): Unique Challenges and Treatment Options.

[12] Hunt L.M., Arar N.H. (2001). An analytical framework for contrasting patient and provider views of the process of chronic disease management. Med Anthropol. Q..

[13] Hunt L.M., Arar N.H., Larme A.C., Rankin S.H., Anderson R.M. (1998). Contrasting patient and practitioner perspectives in type 2 diabetes management. West. J. Nurs. Res..

[14] Nelson S., Slusar M.B., Albert J.M., Riedy C.A. (2017). Do baby teeth really matter? Changing parental perception and increasing dental care utilization for young children. Contemp. Clin. Trials.

[15] Harrison M.S., Harrison R.L. (2007). A conceptual model of parental behavior change following a child’s dental general anesthesia procedure. Pediatric Dent..

[16] Chi D.L., Masterson E.E., Carle A.C., Mancl L.A., Coldwell S.E. (2014). Socioeconomic status, food security, and dental caries in US Children: Mediation analyses of data from the national health and nutrition examination survey, 2007–2008. Am. J. Public Health.

[17] Malterud K., Siersma V.D., Guassora A.D. (2016). Sample size in qualitative interview studies: Guided by information power. Qual. Health Res..

[18] Frank J., Abel T., Campostrini S., Cook S., Lin V.K., McQueen D.V. (2020). The social determinants of health: Time to re-think?. Int. J. Environ. Res. Public Health.

